# Poly[[[μ_3_-*N*′-(carboxymethyl)ethylene­di­amine-*N*,*N*,*N*′-triacetato]dysprosium(III)] trihydrate]

**DOI:** 10.1107/S1600536810041784

**Published:** 2010-10-23

**Authors:** Xiaomei Zhuang, Qingping Long, Jun Wang

**Affiliations:** a Zhongshan Polytechnic, Zhongshan, Guangdong 528404, People’s Republic of China

## Abstract

In the title coordination polymer, {[Dy(C_10_H_13_N_2_O_8_)]·3H_2_O}_*n*_, the dysprosium(III) ion is coordinated by two N atoms and six O atoms from three different (carb­oxy­meth­yl)ethyl­ene­diamine­triacetate ligands in a distorted square-anti­prismatic geometry. The ligands connect the metal atoms, forming layers parallel to the *ab* plane. O—H⋯O hydrogen bonds further assemble adjacent layers into a three-dimensional supra­molecular network.

## Related literature

For general background to the topologies and potential applications of metal coordination polymers, see: Benelli & Gatteschi (2002 ▶). For related structures, see: Wang *et al.* (2007 ▶); You & Ng (2007 ▶); Sakagami *et al.* (1999 ▶); Templeton *et al.* (1985 ▶); Vikram & Sivasankar (2008 ▶).
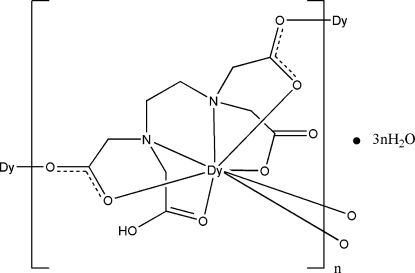


## Experimental

### 

#### Crystal data

[Dy(C_10_H_13_N_2_O_8_)]·3H_2_O
*M* *_r_* = 505.77
Orthorhombic, 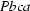

*a* = 13.3835 (5) Å
*b* = 13.0127 (4) Å
*c* = 18.6943 (7) Å
*V* = 3255.7 (2) Å^3^
*Z* = 8
Mo *K*α radiation
μ = 4.65 mm^−1^
*T* = 296 K
0.25 × 0.19 × 0.18 mm

#### Data collection

Bruker APEXII area-detector diffractometer
Absorption correction: multi-scan (*SADABS*; Bruker, 2004 ▶) *T*_min_ = 0.389, *T*_max_ = 0.488
19825 measured reflections
3192 independent reflections
2230 reflections with *I* > 2σ(*I*)
*R*_int_ = 0.034

#### Refinement

*R*[*F* ^2^ > 2σ(*F* ^2^)] = 0.024
*wR*(*F* ^2^) = 0.061
*S* = 1.07
3192 reflections
217 parameters
H-atom parameters constrained
Δρ_max_ = 0.74 e Å^−3^
Δρ_min_ = −0.69 e Å^−3^

### 

Data collection: *APEX2* (Bruker, 2004 ▶); cell refinement: *SAINT* (Bruker, 2004 ▶); data reduction: *SAINT*; program(s) used to solve structure: *SHELXS97* (Sheldrick, 2008 ▶); program(s) used to refine structure: *SHELXL97* (Sheldrick, 2008 ▶); molecular graphics: *SHELXTL* (Sheldrick, 2008 ▶); software used to prepare material for publication: *SHELXTL*.

## Supplementary Material

Crystal structure: contains datablocks I, global. DOI: 10.1107/S1600536810041784/rz2501sup1.cif

Structure factors: contains datablocks I. DOI: 10.1107/S1600536810041784/rz2501Isup2.hkl

Additional supplementary materials:  crystallographic information; 3D view; checkCIF report

## Figures and Tables

**Table 1:** Hydrogen-bond geometry (Å, °)

*D*—H⋯*A*	*D*—H	H⋯*A*	*D*⋯*A*	*D*—H⋯*A*
O1—H1⋯O3^i^	0.82	1.69	2.504 (5)	172
O1*W*—H2*W*⋯O6^ii^	0.85	2.17	2.920 (5)	148
O1*W*—H1*W*⋯O3^iii^	0.84	2.10	2.925 (5)	165
O2*W*—H3*W*⋯O3*W*^iv^	0.83	2.04	2.813 (6)	154
O2*W*—H4*W*⋯O1*W*^v^	0.84	2.09	2.844 (6)	150
O3*W*—H6*W*⋯O2^vi^	0.85	2.56	3.141 (5)	127

Symmetry codes: (i) 
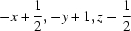
; (ii) 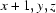; (iii) 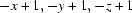; (iv) 
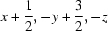
; (v) 
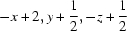
; (vi) 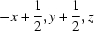.

**Fig. 1.**
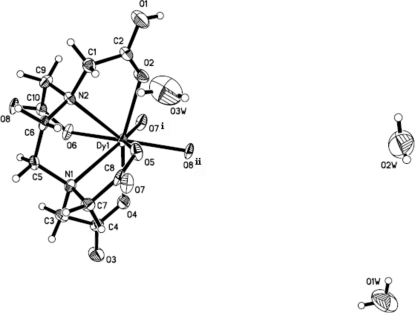
The asymmetric unit of the title compound, with displacement ellipsoids drawn at the 50% probability level. Symmetry codes: (ii) 1/2-x, -1/2+y, z; (iii) 1/2+x, y, 1/2-z.

**Fig. 2.**
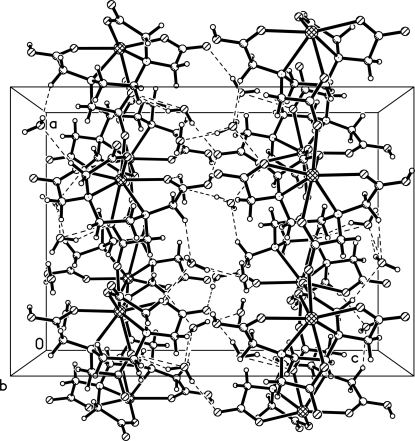
Crystal packing of the title compound viewed along the b axis. Intermolecular hydrogen bonds are shown as dashed lines.

## supplementary crystallographic information

### Comment

The design and construction of metal coordination polymers based on metal ions and multifunctional bridging ligands is of great research interest due to their intriguing topologies and potential applications as functional materials (Benelli & Gatteschi, 2002). The flexible ethylenediaminetetraacetato ligand possessing variable coordination modes to bind to metal ions, provides unique opportunities for the construction of unusual networks. Recently, some mono- and polynuclear Dy complexes of this ligand have been reported (Wang *et al.*, 2007; You & Ng, 2007; Sakagami *et al.*, 1999; Templeton *et al.*, 1985; Vikram & Sivasankar, 2008). Herein, we report the structure of the new polynuclear dysprosium complex, {[Dy(C_10_H_9_N_2_O_8_)].3H_2_O}_n_.

In the structure of the title compound, the dysprosium(III) metal displays a distorted square antiprism geometry provided by two N atoms from one (carboxymethyl)ethylenediaminetriacetato ligand (HEDTA) and six O atoms from three different HEDTA ligands (Fig. 1). The ligands connect the dysprosium centres to form layers parallel to the *ab* plane. O—H···O hydrogen bonds involving the interstitial water molecules assemble adjacent layers to construct a three-dimensional supramolecular network (Table 1;  Fig. 2).

### Experimental

A mixture of Dy_2_O_3_ (0.189 g, 0.5 mmol), ethylenediaminetetraacetic acid (0.146 g, 0.5 mmol), and H_2_O (10 mL) was sealed in a 20 mL Teflon-lined reactor, which was heated in an oven to 423 K for 36 h and then cooled to room temperature at a rate of 5 K h^-1^. Colourless crystals were obtained in a yield of 46% based on Dy.

### Refinement

All water H atoms were tentatively located in difference density Fourier maps and were refined with O–H distance restraints of 0.85 (2) Å and with U_iso_(H) = 1.5 U_eq_(O). In the last stage of refinement, they were treated as riding on their parent O atoms. All H atoms attached to C atoms were fixed geometrically and treated as riding with C—H = 0.97 Å, and *U*_iso_(H) = 1.2*U*_eq_(C).

### Crystal data

**Table:**

[Dy(C_10_H_13_N_2_O_8_)]·3H_2_O	*F*(000) = 1976
*M**_r_* = 505.77	*D*_x_ = 2.064 Mg m^−^^3^
Orthorhombic, *P**b**c**a*	Mo *K*α radiation, λ = 0.71073 Å
Hall symbol: -P 2ac 2ab	Cell parameters from 4800 reflections
*a* = 13.3835 (5) Å	θ = 1.4–28.0°
*b* = 13.0127 (4) Å	µ = 4.65 mm^−^^1^
*c* = 18.6943 (7) Å	*T* = 296 K
*V* = 3255.7 (2) Å^3^	Block, colourless
*Z* = 8	0.25 × 0.19 × 0.18 mm

### Data collection

**Table:**

Bruker APEXII area-detector diffractometer	3192 independent reflections
Radiation source: fine-focus sealed tube	2230 reflections with *I* > 2σ(*I*)
graphite	*R*_int_ = 0.034
φ and ω scan	θ_max_ = 26.0°, θ_min_ = 2.2°
Absorption correction: multi-scan (*SADABS*; Sheldrick, 2008)	*h* = −13→16
*T*_min_ = 0.389, *T*_max_ = 0.488	*k* = −16→16
19825 measured reflections	*l* = −22→20

### Refinement

**Table:**

Refinement on *F*^2^	Primary atom site location: structure-invariant direct methods
Least-squares matrix: full	Secondary atom site location: difference Fourier map
*R*[*F*^2^ > 2σ(*F*^2^)] = 0.024	Hydrogen site location: inferred from neighbouring sites
*wR*(*F*^2^) = 0.061	H-atom parameters constrained
*S* = 1.07	*w* = 1/[σ^2^(*F*_o_^2^) + (0.0239*P*)^2^ + 3.1993*P*] where *P* = (*F*_o_^2^ + 2*F*_c_^2^)/3
3192 reflections	(Δ/σ)_max_ = 0.001
217 parameters	Δρ_max_ = 0.74 e Å^−^^3^
0 restraints	Δρ_min_ = −0.69 e Å^−^^3^

### Special details

**Table:**

Geometry. All esds (except the esd in the dihedral angle between two l.s. planes) are estimated using the full covariance matrix. The cell esds are taken into account individually in the estimation of esds in distances, angles and torsion angles; correlations between esds in cell parameters are only used when they are defined by crystal symmetry. An approximate (isotropic) treatment of cell esds is used for estimating esds involving l.s. planes.
Refinement. Refinement of F^2^ against ALL reflections. The weighted R-factor wR and goodness of fit S are based on F^2^, conventional R-factors R are based on F, with F set to zero for negative F^2^. The threshold expression of F^2^ > 2sigma(F^2^) is used only for calculating R-factors(gt) etc. and is not relevant to the choice of reflections for refinement. R-factors based on F^2^ are statistically about twice as large as those based on F, and R- factors based on ALL data will be even larger.

### Fractional atomic coordinates and isotropic or equivalent isotropic displacement parameters (Å^2^)

**Table:**

	*x*	*y*	*z*	*U*_iso_*/*U*_eq_	
Dy1	0.195820 (13)	0.546220 (11)	0.248589 (10)	0.01538 (8)	
N1	0.1266 (2)	0.6990 (2)	0.32682 (16)	0.0170 (7)	
N2	0.0619 (2)	0.6517 (2)	0.17784 (16)	0.0167 (7)	
C4	0.1889 (3)	0.5806 (3)	0.4196 (2)	0.0220 (10)	
C3	0.1109 (3)	0.6609 (3)	0.4002 (2)	0.0219 (9)	
H3A	0.0448	0.6310	0.4040	0.026*	
H3B	0.1149	0.7178	0.4335	0.026*	
C2	0.1622 (3)	0.5832 (3)	0.0792 (2)	0.0245 (10)	
C1	0.0980 (3)	0.6704 (3)	0.1040 (2)	0.0231 (10)	
H1A	0.1362	0.7337	0.1027	0.028*	
H1B	0.0414	0.6779	0.0721	0.028*	
C6	0.0396 (3)	0.7507 (3)	0.2145 (2)	0.0187 (9)	
H6A	−0.0229	0.7778	0.1964	0.022*	
H6B	0.0918	0.7998	0.2033	0.022*	
C5	0.0323 (3)	0.7387 (3)	0.2951 (2)	0.0195 (9)	
H5A	0.0166	0.8048	0.3163	0.023*	
H5B	−0.0217	0.6918	0.3064	0.023*	
O2	0.1990 (2)	0.5221 (2)	0.12204 (15)	0.0305 (8)	
O4	0.2355 (2)	0.5364 (2)	0.36996 (14)	0.0258 (7)	
O3	0.2014 (2)	0.5610 (2)	0.48468 (15)	0.0375 (8)	
O1	0.1763 (3)	0.5794 (3)	0.01143 (16)	0.0444 (9)	
H1	0.2118	0.5300	0.0018	0.067*	
C8	0.2652 (3)	0.7845 (3)	0.2596 (2)	0.0187 (9)	
C7	0.2041 (3)	0.7799 (3)	0.3277 (2)	0.0204 (9)	
H7A	0.2487	0.7677	0.3677	0.024*	
H7B	0.1722	0.8459	0.3352	0.024*	
O5	0.2750 (2)	0.70278 (19)	0.22414 (15)	0.0228 (6)	
C10	−0.0465 (3)	0.5264 (3)	0.2434 (2)	0.0180 (9)	
C9	−0.0296 (3)	0.5873 (3)	0.1748 (2)	0.0219 (9)	
H9A	−0.0243	0.5399	0.1349	0.026*	
H9B	−0.0870	0.6312	0.1663	0.026*	
O6	0.03078 (19)	0.4997 (2)	0.27769 (15)	0.0218 (6)	
O7	0.30708 (18)	0.86679 (19)	0.24311 (14)	0.0243 (7)	
O8	−0.13277 (19)	0.50137 (19)	0.26058 (14)	0.0216 (6)	
O1W	0.9746 (4)	0.4386 (3)	0.4225 (2)	0.0840 (14)	
H2W	0.9668	0.4475	0.3780	0.126*	
H1W	0.9200	0.4478	0.4441	0.126*	
O2W	0.8853 (3)	0.7777 (3)	0.0541 (2)	0.0798 (13)	
H3W	0.8606	0.7769	0.0133	0.096*	
H4W	0.9063	0.8364	0.0649	0.096*	
O3W	0.3282 (4)	0.7889 (3)	0.0831 (2)	0.0966 (16)	
H6W	0.2814	0.8319	0.0909	0.145*	
H5W	0.3833	0.8247	0.0785	0.145*	

### Atomic displacement parameters (Å^2^)

**Table:**

	*U*^11^	*U*^22^	*U*^33^	*U*^12^	*U*^13^	*U*^23^
Dy1	0.01199 (11)	0.01112 (10)	0.02303 (12)	0.00043 (6)	0.00005 (9)	−0.00001 (9)
N1	0.0157 (17)	0.0155 (15)	0.0197 (18)	0.0009 (14)	−0.0011 (14)	0.0026 (14)
N2	0.0193 (18)	0.0153 (16)	0.0156 (17)	0.0010 (14)	0.0044 (14)	0.0002 (14)
C4	0.023 (2)	0.020 (2)	0.022 (2)	0.0000 (18)	−0.0041 (19)	0.0008 (18)
C3	0.025 (2)	0.025 (2)	0.016 (2)	−0.0007 (19)	0.0048 (18)	−0.0006 (18)
C2	0.024 (2)	0.029 (2)	0.020 (2)	0.002 (2)	0.005 (2)	−0.0044 (19)
C1	0.024 (2)	0.023 (2)	0.022 (2)	0.0052 (19)	0.0024 (18)	0.0024 (18)
C6	0.015 (2)	0.0148 (19)	0.027 (2)	0.0073 (17)	−0.0028 (18)	0.0023 (17)
C5	0.017 (2)	0.0167 (19)	0.025 (2)	0.0043 (17)	0.0026 (18)	−0.0020 (17)
O2	0.042 (2)	0.0291 (16)	0.0204 (16)	0.0182 (14)	0.0014 (14)	−0.0032 (13)
O4	0.0253 (17)	0.0287 (16)	0.0233 (16)	0.0092 (14)	−0.0022 (14)	0.0028 (13)
O3	0.046 (2)	0.048 (2)	0.0180 (16)	0.0207 (16)	−0.0026 (14)	0.0043 (15)
O1	0.059 (2)	0.048 (2)	0.0260 (18)	0.0262 (18)	0.0108 (16)	0.0033 (16)
C8	0.0086 (18)	0.0125 (18)	0.035 (3)	0.0022 (15)	−0.0059 (18)	0.0031 (18)
C7	0.023 (2)	0.0147 (19)	0.023 (2)	−0.0004 (17)	−0.0021 (18)	−0.0029 (17)
O5	0.0212 (15)	0.0139 (14)	0.0332 (16)	−0.0004 (12)	0.0088 (13)	−0.0013 (12)
C10	0.017 (2)	0.0103 (16)	0.026 (2)	0.0009 (15)	−0.0007 (19)	−0.0050 (17)
C9	0.017 (2)	0.025 (2)	0.023 (2)	−0.0019 (18)	−0.0044 (18)	0.0021 (18)
O6	0.0114 (14)	0.0188 (13)	0.0352 (16)	0.0000 (12)	−0.0021 (13)	0.0088 (13)
O7	0.0177 (15)	0.0116 (12)	0.0437 (18)	−0.0019 (11)	0.0034 (14)	0.0005 (14)
O8	0.0077 (14)	0.0187 (12)	0.0383 (18)	−0.0011 (11)	0.0011 (12)	0.0039 (13)
O1W	0.109 (4)	0.097 (3)	0.046 (3)	−0.014 (3)	0.024 (3)	0.002 (2)
O2W	0.062 (3)	0.113 (4)	0.064 (3)	0.018 (3)	−0.004 (2)	0.017 (3)
O3W	0.127 (5)	0.084 (3)	0.079 (3)	0.007 (3)	0.011 (3)	−0.003 (3)

### Geometric parameters (Å, °)

**Table:**

Dy1—O4	2.334 (3)	C6—C5	1.518 (6)
Dy1—O7^i^	2.337 (3)	C6—H6A	0.9700
Dy1—O5	2.342 (3)	C6—H6B	0.9700
Dy1—O6	2.354 (3)	C5—H5A	0.9700
Dy1—O8^ii^	2.373 (3)	C5—H5B	0.9700
Dy1—O2	2.387 (3)	O1—H1	0.8200
Dy1—N2	2.617 (3)	C8—O7	1.248 (4)
Dy1—N1	2.636 (3)	C8—O5	1.260 (4)
N1—C3	1.473 (4)	C8—C7	1.513 (5)
N1—C7	1.477 (5)	C7—H7A	0.9700
N1—C5	1.487 (5)	C7—H7B	0.9700
N2—C1	1.482 (5)	C10—O8	1.243 (4)
N2—C9	1.485 (5)	C10—O6	1.265 (4)
N2—C6	1.488 (5)	C10—C9	1.524 (5)
C4—O4	1.257 (5)	C9—H9A	0.9700
C4—O3	1.254 (5)	C9—H9B	0.9700
C4—C3	1.521 (5)	O7—Dy1^iii^	2.337 (3)
C3—H3A	0.9700	O8—Dy1^iv^	2.373 (2)
C3—H3B	0.9700	O1W—H2W	0.8462
C2—O2	1.231 (5)	O1W—H1W	0.8429
C2—O1	1.282 (5)	O2W—H3W	0.8322
C2—C1	1.496 (5)	O2W—H4W	0.8371
C1—H1A	0.9700	O3W—H6W	0.8515
C1—H1B	0.9700	O3W—H5W	0.8763
			
O4—Dy1—O7^i^	89.53 (9)	O2—C2—O1	124.0 (4)
O4—Dy1—O5	97.72 (10)	O2—C2—C1	121.2 (4)
O7^i^—Dy1—O5	150.23 (9)	O1—C2—C1	114.8 (4)
O4—Dy1—O6	88.56 (10)	N2—C1—C2	110.6 (3)
O7^i^—Dy1—O6	74.80 (9)	N2—C1—H1A	109.5
O5—Dy1—O6	133.90 (9)	C2—C1—H1A	109.5
O4—Dy1—O8^ii^	80.61 (10)	N2—C1—H1B	109.5
O7^i^—Dy1—O8^ii^	76.54 (8)	C2—C1—H1B	109.5
O5—Dy1—O8^ii^	76.25 (9)	H1A—C1—H1B	108.1
O6—Dy1—O8^ii^	149.38 (9)	N2—C6—C5	112.4 (3)
O4—Dy1—O2	162.25 (10)	N2—C6—H6A	109.1
O7^i^—Dy1—O2	79.94 (10)	C5—C6—H6A	109.1
O5—Dy1—O2	85.01 (10)	N2—C6—H6B	109.1
O6—Dy1—O2	102.22 (10)	C5—C6—H6B	109.1
O8^ii^—Dy1—O2	83.05 (9)	H6A—C6—H6B	107.9
O4—Dy1—N2	132.53 (9)	N1—C5—C6	112.1 (3)
O7^i^—Dy1—N2	119.38 (9)	N1—C5—H5A	109.2
O5—Dy1—N2	75.81 (10)	C6—C5—H5A	109.2
O6—Dy1—N2	66.98 (9)	N1—C5—H5B	109.2
O8^ii^—Dy1—N2	138.99 (9)	C6—C5—H5B	109.2
O2—Dy1—N2	65.17 (9)	H5A—C5—H5B	107.9
O4—Dy1—N1	65.28 (9)	C2—O2—Dy1	123.5 (3)
O7^i^—Dy1—N1	140.53 (9)	C4—O4—Dy1	125.5 (3)
O5—Dy1—N1	67.10 (9)	C2—O1—H1	109.5
O6—Dy1—N1	74.70 (10)	O7—C8—O5	123.1 (4)
O8^ii^—Dy1—N1	124.40 (9)	O7—C8—C7	119.0 (3)
O2—Dy1—N1	130.94 (9)	O5—C8—C7	117.8 (3)
N2—Dy1—N1	69.15 (9)	N1—C7—C8	113.4 (3)
C3—N1—C7	109.2 (3)	N1—C7—H7A	108.9
C3—N1—C5	111.5 (3)	C8—C7—H7A	108.9
C7—N1—C5	110.7 (3)	N1—C7—H7B	108.9
C3—N1—Dy1	108.2 (2)	C8—C7—H7B	108.9
C7—N1—Dy1	107.3 (2)	H7A—C7—H7B	107.7
C5—N1—Dy1	109.8 (2)	C8—O5—Dy1	125.7 (2)
C1—N2—C9	109.0 (3)	O8—C10—O6	123.8 (4)
C1—N2—C6	110.6 (3)	O8—C10—C9	119.4 (3)
C9—N2—C6	109.9 (3)	O6—C10—C9	116.7 (3)
C1—N2—Dy1	109.4 (2)	N2—C9—C10	112.5 (3)
C9—N2—Dy1	106.7 (2)	N2—C9—H9A	109.1
C6—N2—Dy1	111.0 (2)	C10—C9—H9A	109.1
O4—C4—O3	123.9 (4)	N2—C9—H9B	109.1
O4—C4—C3	118.5 (4)	C10—C9—H9B	109.1
O3—C4—C3	117.6 (4)	H9A—C9—H9B	107.8
N1—C3—C4	110.9 (3)	C10—O6—Dy1	125.4 (2)
N1—C3—H3A	109.5	C8—O7—Dy1^iii^	147.0 (2)
C4—C3—H3A	109.5	C10—O8—Dy1^iv^	144.6 (2)
N1—C3—H3B	109.5	H2W—O1W—H1W	110.2
C4—C3—H3B	109.5	H3W—O2W—H4W	111.4
H3A—C3—H3B	108.1	H6W—O3W—H5W	106.7

Symmetry codes: (i) −*x*+1/2, *y*−1/2, *z*; (ii) *x*+1/2, *y*, −*z*+1/2; (iii) −*x*+1/2, *y*+1/2, *z*; (iv) *x*−1/2, *y*, −*z*+1/2.

### Hydrogen-bond geometry (Å, °)

**Table:**

*D*—H···*A*	*D*—H	H···*A*	*D*···*A*	*D*—H···*A*
O1—H1···O3^v^	0.82	1.69	2.504 (5)	172
O1W—H2W···O6^vi^	0.85	2.17	2.920 (5)	148
O1W—H1W···O3^vii^	0.84	2.10	2.925 (5)	165
O2W—H3W···O3W^viii^	0.83	2.04	2.813 (6)	154
O2W—H4W···O1W^ix^	0.84	2.09	2.844 (6)	150
O3W—H6W···O2^iii^	0.85	2.56	3.141 (5)	127

Symmetry codes: (v) −*x*+1/2, −*y*+1, *z*−1/2; (vi) *x*+1, *y*, *z*; (vii) −*x*+1, −*y*+1, −*z*+1; (viii) *x*+1/2, −*y*+3/2, −*z*; (ix) −*x*+2, *y*+1/2, −*z*+1/2; (iii) −*x*+1/2, *y*+1/2, *z*.

## References

[bb1] Benelli, C. & Gatteschi, D. (2002). *Chem. Rev.* **102**, 2369-2388. 10.1021/cr010303r 12059272

[bb2] Bruker (2004). *APEX2*, *SAINT* and*SADABS* Bruker AXS Inc, Madison, Wisconsin, USA.

[bb3] Sakagami, N., Yamada, Y., Konno, T. & Okamoto, K. (1999). *Inorg. Chim. Acta*, **288**, 7–16.

[bb4] Sheldrick, G. M. (2008). *Acta Cryst.* A**64**, 112–122. 10.1107/S0108767307043930 18156677

[bb5] Templeton, L. K., Templeton, D. H. & Zalkin, A. (1985). *Acta Cryst.* C**41**, 355–358.

[bb6] Vikram, L. & Sivasankar, B. N. (2008). *Ind. J. Chem. Sect. A*, **47**, 25–31.

[bb7] Wang, J., Gao, G., Zhang, Z., Zhang, X. & Wang, Y. (2007). *J. Coord. Chem.* **60**, 2221–2241.

[bb8] You, X.-L. & Ng, S. W. (2007). *Acta Cryst.* E**63**, m1819.

